# Deciphering the epidemic synergy of herpes simplex virus type 2 (HSV-2) on human immunodeficiency virus type 1 (HIV-1) infection among women in sub-Saharan Africa

**DOI:** 10.1186/1756-0500-5-451

**Published:** 2012-08-21

**Authors:** Musie Ghebremichael, Desale Habtzgi, Elijah Paintsil

**Affiliations:** 1 Harvard Medical School and Ragon Institute of MGH, MIT and Harvard, Boston, Massachusetts, USA; 2 Department of Statistics, University of Akron, Akron, Ohio, USA; 3 Departments of Pediatrics and Pharmacology, Yale School of Medicine, New Haven, Connecticut, USA

## Abstract

**Background:**

Herpes Simplex Virus Type 2 (HSV-2) is highly prevalent in regions disproportionately affected by the human immunodeficiency virus (HIV-1) epidemic. The objective of our study was to identify the risk factors of HSV-2 and HIV-1 infections and to examine the association between the two infections.

**Methods:**

The study participants were recruited through a community based cross-sectional study that was conducted from November 2002 to March 2003 in the Moshi urban district of Northern Tanzania. A two-stage sampling design was used in recruiting the study participants. Information on socio-demographics, alcohol use, sexual behaviors, and STIs symptoms were obtained. Blood and urine samples were drawn for testing of HIV-1, HSV-2 and other STIs.

**Results:**

The prevalence of HSV-2 infection among all study participants was 43%. The prevalence rate of HSV-2 among the HIV-negative and HIV-positive women was 40% and 65%, respectively. We found 2.72 times odds of having HIV-1 in an HSV-2 positive woman than in an HSV-2 negative woman. Furthermore, HIV-1 and HSV-2 shared common high-risk sexual behavior factors such as early onset of sexual debut, and testing positive for other STIs.

**Conclusions:**

Our findings suggest that HSV-2 may be both a biological and risk-associated cofactor for HIV-1 acquisition. In resource-limited countries, where both infections are prevalent efforts at symptomatic and diagnostic screening and treatment of HSV-2 should be part of HIV-1 prevention programs.

## Background

The correlate of herpes simplex type 2 (HSV-2) and human immunodeficiency virus type 1 (HIV-1) epidemics is intriguing and has been the subject of several studies. HSV-2 is a common infection, with an estimated prevalence and incidence of 536 and 23.6 million infections, respectively, in 15 – 49-year-olds worldwide [1]. HSV-2 infection is highly prevalent in regions disproportionately affected by the HIV-1 epidemic. Several epidemiological studies have demonstrated higher incidence and prevalence rates of HIV-1 among HSV-2-infected populations and vice versa [2-5]. Sub-Saharan Africa with the highest prevalence of HIV-1 has about 80% prevalence rate of HSV-2 among its adult population [6,7]. The prevalence of HSV-2 in HIV-infected individuals in sub-Saharan Africa is 50 to 90% [8]. Furthermore, there is a high seroprevalence of HSV-2 in communities with high incidence of HIV-1 in developed countries [9,10]. The similarities between the two infections with regard to prevalence rates, risk factors, and geographical distribution cannot be a mere coincidence. If the association between HSV-2 infection and HIV-1 is causal, then the proportion of HIV infections attributable to HSV-2 infection is high, estimated at 25% –35% in sub-Saharan Africa [11]. Thus, the high prevalence of HSV-2 and its interaction with HIV-1 might have played a significant role in the HIV-1 epidemic in sub-Saharan Africa.

Several epidemiological and laboratory studies have suggested a biological plausibility of HSV-2 infection facilitating the acquisition and transmission of HIV-1. Recurrent episodes of HSV-2 may increase the susceptibility to HIV-1 infection in HSV-infected individuals by activating HIV-1 target cells in the genital tract [8,12,13]. In HIV-1-serodiscordant couples, HSV-2-seropositive individuals were at increased risk of acquiring HIV-1 [14,15]. In individuals with HIV-1 and HSV-2 co-infection, symptomatic and asymptomatic reactivation of HSV-2 has been associated with increased plasma HIV-1 viral load and genital shedding of HIV-1 [16]. To establish a relationship between HSV-2 and HIV-1 replication, several randomized controlled trails have tested the effect of HSV-2 suppressive therapy on HIV-1 transmission [14,17-20]. Unfortunately, the effect of HSV-2 suppressive therapy - with acyclovir - on HIV-1 transmission in these studies has been mixed. However, most of these studies demonstrated a reduction in plasma and genital HIV-1 RNA concentrations in co-infected individuals. Acyclovir, used in these trials, has direct anti-HIV effect by inhibiting HIV-1 reverse transcriptase [21,22]. Therefore, it has been argued that the modest effect on HIV replication seen in these trials may not be directly related to HSV-2-HIV-1 interaction. Moreover, the mixed reports might be due to the rather complex nature of the interaction between the two pathogens and the fact that both epidemics have matured and reached saturation making it difficult to tease out the relationship by clinical trials.

We hypothesized that HSV-2 epidemic might have contributed to the evolution of the HIV epidemic in sub-Saharan Africa. To test this hypothesis, we used data from a community-based survey in the Moshi urban district of northern Tanzania. The prevalence of HSV-2 in Tanzania is estimated at 70% [23]. However, in the study cohort, the prevalence of HSV-2 and HIV-1 are 43% and 11%, respectively [24]. The objectives of our study were: 1) to identify shared-risk factors of HSV-2 and HIV-1 infections and 2) to examine the association between HIV-1 and HSV-2 among study participants.

## Methods

### Study design

A detailed description of the study design and characteristics of the study participants has been described previously [24]. In brief, the study participants were recruited through a community based cross-sectional study that was conducted from November 2002 to March 2003 in the Moshi urban district of Northern Tanzania. A two-stage sampling design was used in recruiting the study participants. First, 150 geographic clusters were randomly selected from Moshi and then a number of households were randomly selected from each cluster. A total of 2019 women who were residents of the households and 794 men who were their partners were enrolled into the study. The participants’ socio-demographic, high-risk behavior, biological, and medical characteristics were assessed. The study was approved by the Harvard School of Public Health IRB (HSC Protocol #0108ACOM), University of Maryland IRB (Protocol #05-0031), Kilimanjaro Christian Medical Center Ethics Committee, Research and Ethical Clearance of the Tanzanian National Institute for Medical Research, the Centers for Disease Control and Prevention Institutional Review Board. Written informed consent for participation in the study was obtained from participants.

### Laboratory methods

Blood and urine samples were collected and tested for HIV-1, HSV-2 and other sexually transmitted infections (STIs). HIV-1 infection was determined using HIV enzyme-linked immunosorbent assay (ELISA) (Vironostika HIV Uni-Form II Plus O, Organon, Boxtel, The Netherlands) and reactive samples were confirmed using Wellcozyme HIV-1 ELISA test (Murex 1.2.0, Murex Biotech Ltd., UK). Western blot tests (Bio-Rad Laboratories Ltd., Dartford, UK) were used to confirm discordant ELISA test results. Antibodies to HSV-2 were detected using enzyme immune assay (EIA) according to the manufacturer’s instructions (HerpeSelect 2 ELISA, Focus Technologies, Cypress, CA). Active and past syphilis were diagnosed if the serum was reactive on the Rapid Plasma Reagin card test (Macro-Vue; Becton-Dickinson, Cockeysville, MD) and/or the Treponema Pallidum xHemagglutination Assay (TPHA) (Wecosyph HA; Murex Bio-tech Ltd., UK). Urine samples were tested for Chlamydia, gonorrhea, trichomonas and mycoplasma genitalium by using a real-time multiplex polymerase chain reaction (M-PRC) assay.

### Study measures

Socio-demographic characteristics (e.g., age in years, religion, ethnicity, education), high-risk behaviors (e.g., age at first sex, number of sexual partners in the previous three years, alcohol use, condom use), and biological/medical factors (e.g., circumcision, symptoms of STIs and STIs other than HIV-1/HSV-2) were considered as covariates in our analyses. Alcohol abuse was measured by the CAGE score. An STI’s symptom was defined as having at least one of the following symptoms: abdominal pain, abnormal genital discharge, foul smell in the genital area, excessive genital secretions, swellings in the genital area, itching in the genital area, burning pain on micturition, pain during intercourse, and genital ulcers.

### Statistical methods

We have a clustered binary data with individuals (level-1), i.e., women, nested in geographic clusters (level-2). It is reasonable to assume that measurements from individuals within the same cluster were correlated. Because of the inherent intra-cluster correlation between binary outcomes, statistical modelling of the data using the ordinary logistic regression is not appropriate. Therefore, multilevel generalized linear mixed effects models were employed in assessing the risk factors for both infections and the interplay between them. The correlation among individuals from the same cluster was incorporated using a random intercept at the cluster level. Measurements from individuals within the same cluster were assumed to be independent given the random intercept. Model parameters were estimated via maximum likelihood based on adaptive quadrature [25]. The analysis was performed using the GLIMMIX procedure in SAS [26].

## Results

### Characteristics of study participants by their HSV-2 and HIV-1 status

Among the 1418 women who provided blood samples, 154 and 609 were seropositive for HIV-1 and HSV-2, respectively. The prevalence rate of HSV-2 among the women who tested positive for HIV-1 was 65%, which was 1.63 times higher than the rate of HSV-2 in women who tested negative for HIV-1. Preliminary analysis was done using Pearson’s Chi-square and Fisher’s exact tests to identify the potential risk factors of HSV-2 and HIV-1 infections (Table 1). Table 1 presents participants’ socio-demographic, risk behavior and medical/biological characteristics by their HSV-2 and HIV-1 infection status. The prevalence of both HSV-2 (p < 0.01) and HIV-1 (p = 0.04) was significantly associated with the age of the participant. Religion was significantly associated with HSV-2 (p = 0.05) but not with HIV-1 (p = 0.54). HSV-2 prevalence rate was lower for Protestants compared to that of the other religions/denominations. Number of sexual partners in the previous three years was associated with HIV-1 (p < 0.01) but not with HSV-2 (p = 0.26). HSV-2 and HIV-1 prevalence rates were significantly associated with age at first sex (p < 0.01), with higher rates among women who experienced an early sexual debut. That is, postponing age at first sexual debut was protective for both infections. Having a positive test for STI (e.g., syphilis, chlamydia, gonorrhea, trichomonas and mycoplasma genitalium) was significantly associated with HSV-2 and HIV-1 status (p < 0.01). There was a significant association between having symptoms of STIs at study enrolment and HIV-1 status (p = 0.04). However, the association between symptoms of STIs and HSV-2 status was only marginally significant (p = 0.06). HSV-2 status was significantly associated with HIV-1 status (p < 0.01). Female circumcision, alcohol use and condom use were not associated with either HIV-1 or HSV-2 infection status.

**Table 1:** Herpes simplex virus type-2 and HIV-1 prevalence by socio-demographic, high risk behavior, biological and medical factors among women in Moshi district, northern Tanzania

**Variables**	**HSV-2 (N = 1418)**			**HIV-1 (N = 1418)**		
	**Negative** ** N (%)**	**Positive** ** N (%)**	**P-value**	**Negative** ** N (%)**	**Positive** ** N (%)**	**P-value**
*Age*			<0.01			0.04
20-24	354 (44)	99 (16)		419 (33)	34 (22)	
25-29	205 (25)	140 (23)		303 (24)	42 (27)	
30-34	119 (15)	140 (23)		228 (18)	31 (20)	
35-39	78 (10)	120 (20)		168 (13)	30 (20)	
≥ 40	52 (6)	109 (18)		144 (12)	17 (11)	
*Ethnicity*			0.18			0.56
Chaga	438 (54)	301 (49)		665 (53)	74 (48)	
Pare	98 (12)	77 (13)		154 (12)	21 (14)	
Other	272 (34)	231 (38)		44 4(35)	59 (38)	
*Religion*			0.05			0.54
Muslim/other	255 (32)	229 (38)		430 (34)	54 (35)	
Catholic	332 (41)	237 (39)		503 (40)	66 (43)	
Protestant	221 (27)	143 (23)		330 (26)	34 (22)	
*Education*			0.52			0.34
Pre-secondary	622 (77)	477 (78)		975 (77)	124 (81)	
Secondary and above	187 (23)	132 (22)		289 (23)	30 (19)	
*Alcohol abuse*			0.71			0.82
No	296 (84)	291 (85)		508 (85)	79 (84)	
Yes	55 (16)	50 (15)		90 (15)	15 (16)	
*Number of partners†*			0.26			<0.01
1	587 (90)	492 (88)		967 (91)	112 (79)	
2+	64 (10)	66 (12)		100 (9)	30 (21)	
*Age at first sex*			<0.01			<0.01
<18	222 (28)	248 (42)		392 (32)	78 (52)	
18-19	215 (27)	159 (27)		339 (27)	35 (23)	
20+	353 (45)	188 (31)		503 (41)	38 (25)	
*Condom use‡*			0.85			0.27
Never	500 (77)	429 (77)		826 (78)	103 (75)	
Sometimes	133 (21)	115 (21)		218 (20)	30 (22)	
Often/always	11 (2)	12 (2)		18 (2)	5 (3)	
*Female circumcision*			0.06			0.98
No	624 (77)	442 (73)		950 (75)	116 (75)	
Yes	185 (23)	166 (27)		313 (25)	38 (25)	
Test positive STIs*			<0.01			<0.01
Negative	715 (88)	500 (82)		1098 (87)	117 (76)	
Positive	94 (12)	109 (18)		166 (13)	37 (24)	
*STIs Symptoms*			0.06			0.04
No	498 (71)	392 (66)		797 (70)	93 (62)	
Yes	204 (29)	201 (34)		347 (30)	58 (38)	
*HSV-2*			-			<0.01
No	-	-		755 (60)	54 (35)	
Yes	-	-		509 (40)	100 (65)	

*Syphilis, Chlamydia, gonorrhea, trichomonas and mycoplasma genitalium.

### Multivariate analysis: risk factors for HSV-2 and HIV-1 and effect of HSV-2 on HIV-1

**Figure 1:**
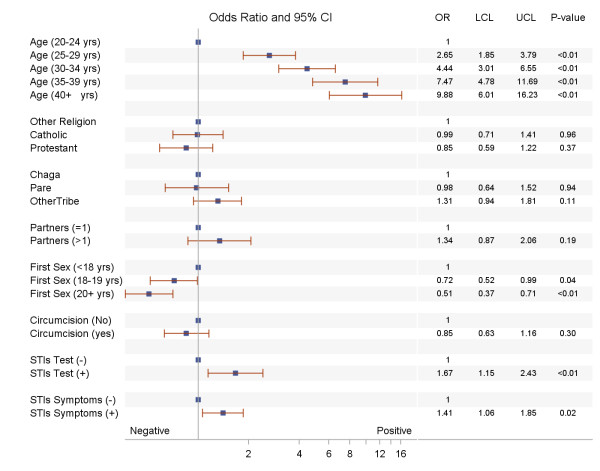
Risk factors for HSV-2 among women in Moshi district, northern Tanzania.

**Figure 2:**
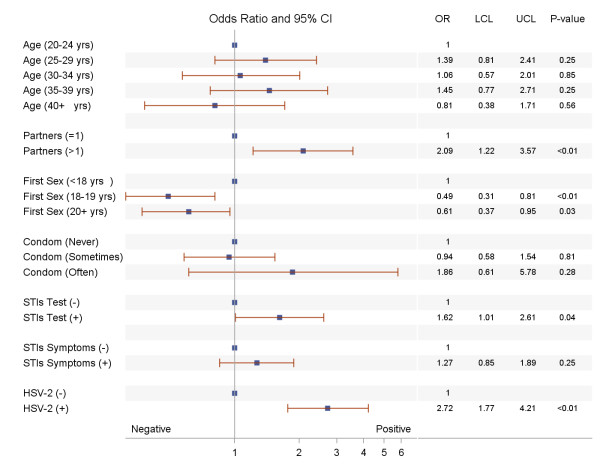
Risk factors for HIV-1 among women in Moshi district, northern Tanzania.

Multivariate analysis using multilevel generalized linear mixed effects models - with women nested in geographic clusters - were fitted. All the variables that were found to be significantly associated with HIV-1 and HSV-2 at 0.30 levels in the univariate analysis were included as covariates in the multivariate analysis. The results of the multivariate analyses are reported in Figures 1, 2.

The adjusted results in Figures 1, 2 indicate that older women were more likely to have HSV-2 compared to younger women. For example, the odds of having HSV-2 in a woman 40 years or older was 9.88 times higher than that of woman between 20 and 24 years (OR = 9.88; 95%, CI: 6.01-16.23). The effects of religion, ethnicity, number of sexual partners and female circumcision on HSV-2 were not significant. However, age at sexual onset was significantly associated with HSV-2. Women who started their sexual debut before 18 years were more likely to have HSV-2 that those who began to have sex at 18 to 19 years or age 20 and older (OR = 0.72, 95% CI = 0.52-0.99; OR = 0.51, 95% CI = 0.37-0.71). Testing positive for other STIs (OR = 1.67, 95% CI = 1.15-2.43) and having STI symptoms (OR = 1.41, 95% CI = 1.06-1.85) were associated with a higher prevalence of HSV-2.

Women who tested positive for HSV-2 had a higher prevalence of HIV-1. The odds of having HIV-1 in HSV-2 positive women were 2.72 times higher than that of women who tested negative for HSV-2 (OR = 2.72; 95%, CI: 1.77-4.21). In contrast to HSV-2, HIV-1 prevalence did not significantly vary by the age of the woman. However, age at sexual debut was significantly associated with HIV-1 as observed with HSV-2. Women who had first sex at 18 to 19 years or age 20 and older were less likely to have HIV-1 than women who had first sex before age 18 (OR = 0.49, 95% CI = 0.31-0.81; OR = 0.61, 95% CI = 0.37-0.95). Moreover, women who had two or more partners in the previous three years (OR = 2.09; 95% CI: 1.22-3.57) were more likely to have HIV-1 compared with women who had one partner. Furthermore, testing positive for other STIs (OR = 1.62, 95% CI = 1.01-2.61) were associated with high prevalence of HIV-1.

## Discussion

The prevalence of HSV-2 infection among all study participants was 43%. The prevalence rate of HSV-2 among the HIV-negative and HIV-positive women was 40% and 65%, respectively. These rates are comparable to the rates reported in other studies from sub-Saharan Africa [23]. We found 2.72 times odds of having HIV-1 in an HSV-2 seropositive woman than in an HSV-2 seronegative woman. Our finding is consistent with a previous meta-analyses of cohort studies that showed that HSV-2 infection led to two to three times increase in risk of acquiring HIV-1 [5,27,28]. Two recent cohort studies from Uganda and Zimbabwe reported hazards ratios of HIV-1 acquisition of 2.8 and 4.4 for prevalent HSV-2 infection, respectively, and 4.6 and 8.6 for incident infection, respectively [29].

Consistent with other studies from the sub-region [30,31], we found a direct association between HSV-2 and HIV-1 infections. Our finding of HSV-2 as a significant predictor for HIV-1 infection coupled with findings from other investigators that HSV-2 acquisition generally precedes HIV-1 seroconversion [30] support our hypothesis that HSV-2 epidemic might have facilitated the HIV-1 epidemic in sub-Saharan Africa. The interplay between HIV-1 and HSV-2 infections is known to be bi-directional and complex. From our study and that of others, HSV-2 seems to serve as both a high-risk and biological cofactor for HIV-1 acquisition.

HIV-1 and HSV-2 shared common high-risk sexual behavior factors such as early onset of sexual debut, and testing positive for other STIs. Is it possible that the association between HIV-1 acquisition and HSV-2 is confounded by high-risk sexual behaviors placing individuals at risk for both viruses? This concept is attractive and may help explain the inconsistent results of efficacy trials of HSV-2 suppression for prevention of HIV acquisition. However, our data will argue to the contrary. The higher prevalence of HSV-2 and its predominance in older women suggest that HSV epidemic predates that of HIV. Moreover, it is well known that infection with one STI may increase the probability of acquiring or transmitting another STI. These could be the potential reasons for the high prevalence of HIV-1 in our study among women who were HSV-2 seropositive. The increased risk of HSV-2 and HIV-1 associated with an early age at first sex may be partly due to biological, behavioral, and socio-economic predispositions [24].

HSV-2 may serve as a biological cofactor for HIV-1 acquisition. HSV-2 creates potential biological bridges for HIV acquisition; by erosion of the epithelial barrier through which HIV-1 can enter and by causing localized inflammation that leads to the recruitment of new HIV-1 susceptible targets such as activated CD4+ T-cells and macrophages that live in the sub- epithelium [32,33]. Furthermore, some HSV-2 proteins have been reported to directly interact *in vitro* with HIV-1 long terminal repeat (LTR) region to up-regulate HIV-1 replication through NF-κB activation [34]. The above biological phenomena lead to increase HIV-1 viral load in plasma and genital secretions of individuals with HIV-1 and HSV-2 co-infection during symptomatic and asymptomatic reactivation of HSV-2 [16].

The present study has several strengths compared to previous studies; it has a large sample size and used laboratory-confirmed STIs in a population based sample. Most studies of the prevalence of HSV-2 and its role as a risk factor for HIV have been carried out in particular groups that are not representative of the overall population, such as STD or antenatal clinic attendees, blood donors, or men who have sex with men [35]. However, our study had some limitations. A strong case for causality cannot be made given the cross-sectional nature of the study; however, our findings reiterate the need for further research. Longitudinal studies, taking into consideration the anti-HIV effect of acyclovir, are needed to elucidate the nature of the relationship between HSV-2 and HIV-1 infections.

## Conclusions

In conclusion, we found a significant association and synergy between HSV-2 and HIV-1 infections among study participants. HSV-2 may be a biological and risk cofactor for HIV-1 acquisition. In resource-limited countries, where both infections are prevalent efforts at symptomatic and diagnostic screening and treatment of HSV-2 could be part of the preventive measures for HIV-1. Furthermore, our study identified specific high-risk behaviors – early onset of sexual debut, multiple sexual partners and presence of other STIs – that could be used in crafting messages for HIV-1 prevention programs.

## Competing interests

The authors declare that they have no competing interests.

## Authors’ contributions

MG designed the study and analyzed the data. MG and EP wrote the article. DH helped with the revision of the manuscript. All authors read and approved the final manuscript.
